# Synopsis of *Falsocis* Pic (Coleoptera, Ciidae), new species, new records and an identification key

**DOI:** 10.3897/zookeys.145.1895

**Published:** 2011-11-04

**Authors:** Cristiano Lopes-Andrade, John F. Lawrence

**Affiliations:** 1 Departamento de Biologia Animal, Universidade Federal de Viçosa, 36570-000, Viçosa, Minas Gerais, Brazil; 2 Australian National Insect Collection, CSIRO Ecosystem Sciences, GPO Box 1700, Canberra, ACT 2601, Australia

**Keywords:** Minute tree-fungus beetles, Ciinae, Neotropical, Tenebrionoidea

## Abstract

Three new species of *Falsocis* Pic are described: *Falsocis aquilonius* **sp. n.** from Panamá, Costa Rica and Colombia, *Falsocis egregius* **sp. n.** from a single locality in northern Brazil and *Falsocis occultus* **sp. n.** from two localities in southeastern and southern Brazil. New records, comparative notes and an identification key for male and female specimens of *Falsocis* species are also provided.

## Introduction

*Falsocis* Pic, 1916 is a distinctive but relatively uniform genus of Neotropical Ciidae, recently redescribed by Lopes-Andrade (2007) and currently including two species: *Falsocis opacus* Pic, 1916, from French Guyana and northern Brazil; and *Falsocis brasiliensis* Lopes-Andrade, 2007, an endangered species occurring in small remnants of the Brazilian Atlantic Forest in the states of Minas Gerais, Espírito Santo and Bahia. *Falsocis* species are not frequently collected and few specimens are known from museum collections.

The aims of this work are to provide new records and comparative notes on *Falsocis*, descriptions of three additional species of the genus and an updated identification key for the species.

## Material and methods

Slide preparations of male terminalia, and methods for photographing slide preparations and specimens, followed mostly Lopes-Andrade (2011), except for the stereosmicroscope and camera used, a Zeiss Stemi 2000-C and a Canon EOS 1000D attached, respectively. Terms for external morphology of ciids are explained and discussed by Lopes-Andrade and Lawrence (2005) and Lopes-Andrade (2008). It must be emphasized that “tegmen”, as used here, is synonymous with “paramere”, “parameral piece” or “apicale” and not with the same term as used in Cucujoidea, Chrysomeloidea and Curculionoidea, which is essentially synonymous with phallobase or basal piece (see Lawrence and Lopes-Andrade 2010). The terms “low”, “medium” and “high” (or “minor”, “intermediate” and “major”; Scott 1926) refers to the conspicuousness of secondary sexual characters in male head and pronotum of ciids. Low males are the ones with the weakest horns and/or tubercles, being barely discernible from females. In medium males, these features are conspicuous but not developed at their most. High males have the most prominent horns and/or tubercles. Such variation in male development is common in ciids and usually seen as two categorical forms, low and high, in species known from few localities and/or small series (e.g. *Cis pickeri* Lopes-Andrade et al. and *Xylographus seychellensis* Scott; see Lopes-Andrade et al. 2009; Scott 1926) and seen as a continuum in widely distributed and frequently collected ciids (e.g. *Ceracis cornifer* Mellié; Lopes-Andrade pers. obs.). The new species described here were compared to named specimens of both described *Falsocis* species, and all type specimens were examined. Images of all described and new species are provided, but images of male terminalia of *Falsocis opacus* and *Falsocis brasiliensis* are available in Lopes-Andrade (2007) and not repeated here. The identification key was elaborated to work for both male and female specimens. However, it is not advisable to identify any *Falsocis* based on female specimens alone, since secondary sexual characters and male terminalia are important features for recognizing species.

Range, mean and standard deviation are given for measurements and ratios. Measurements of antennomeres were taken from the holotypes. The following abbreviations are used for measurements and ratios: CL, length of the antennal club; EL, elytral length (median length from base of scutellum to elytral apex); EW, greatest elytral width; FL, length of the antennal funicle; GD, greatest depth of the body; PL, pronotal length along midline (including the anterior pronotal plate in males); PW, greatest pronotal width; TL, total length (EL+PL; head not included). The ratio GD/EW was taken as an indication of degree of convexity; TL/EW indicates degree of body elongation. Values for TL and TL/EW of the type series of *Falsocis brasiliensis* and the unique available male *Falsocis opacus* are also provided here, since in Lopes-Andrade (2007) TL included the length of the head seen from above. Each description is based on the holotype, which is a fully pigmented male. Differences among paratypes are given in the section on “Variation”, together with standard measurements and ratios of the type series.

The distribution map (Fig. 41) was created using latitude and longitude coordinates estimated by tracking localities in the freeware Google Earth 6.0.3 (Google Earth 2011) and plotting them in a map using the freeware DIVA-GIS 7.3.0 (Hijmans et al. 2001).

**The following acronyms are used in this paper:**

ANICAustralian National Insect Collection, CSIRO Entomology (Canberra, Australia)


CMNCanadian Museum of Nature (Ottawa, Ontario, Canada)


CMNHCarnegie Museum of Natural History (Pittsburgh, Pennsylvania, USA)


CNCICanadian National Collection of Insects (Ottawa, Ontario, Canada)


FMNHField Museum of Natural History (Chicago, Illinois, USA)


LAPCCristiano Lopes-Andrade Private Collection (Viçosa, MG, Brazil)


MNHNMuséum National d’Histoire Naturelle (Paris, France)


MTDMuseum für Tierkunde Dresden (Dresden, Germany)


## Taxonomy

### Falsocis aquilonius

Lopes-Andrade & Lawrence sp. n.

urn:lsid:zoobank.org:act:01613AEF-7593-43F1-B83C-CB7F8A13AF21

http://species-id.net/wiki/Falsocis_aquilonius

Figs 1 –9 41

#### Type locality.

Cerro Campana, in the province of Panamá, Panama (8°26'N, 81°17'W).

#### Etymology.

The specific epithet is from the Latin“aquilonius" (adjective), which means “from the North", in reference to its occurrence at the northernmost locality for *Falsocis* species.

#### Diagnosis.

Epipleura enlarged posteriorly, but just slightly explanate (Figs 2–3), not or barely visible from above, with external margin simple (not crenulate). Pronotum with lateral margins visible for their entire lengths from above. Male abdominal sex patch small with a diameter of one-fifth the length of the first abdominal ventrite.

#### Description.

**Figures 1–6.**
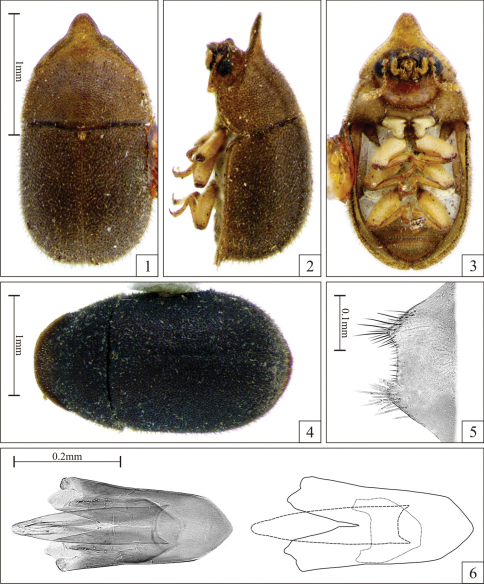
*Falsocis aquilonius* Lopes-Andrade & Lawrence, sp. n., male holotype **1–3**, shown in the same scale, female paratype from Puntarenas, Costa Rica **4** and slide preparation of male terminalia of a paratype **5–6**. **1** Dorsal view **2** Lateral view **3** Ventral view **4** Dorsal view **5** Eighth sternite **6** Aedeagus (left) and gross outline of aedeagus (right) showing tegmen (continuous line), penis (dashed line) and basal piece (dotted line).

Male holotype (Figs 1–3), measurements in mm: TL 2.21; PL 0.95; PW 1.11; EL 1.26; EW 1.16; GD 0.89. Ratios: PL/PW 0.86; EL/EW 1.09; EL/PL 1.33; GD/EW 0.77; TL/EW 1.91. Body oblong, strongly convex, mostly yellowish brown; coxae and femora pale yellow; mesoventrite, metaventrite and first abdominal ventrite whitish. Head not visible from above; frontoclypeal ridge slightly raised, bearing two very short, barely pronounced tubercles; disc slightly convex, closely and coarsely punctate, glabrous; in between punctures finely granulate. Eyes coarsely facetted; greatest eye width 0.15mm. Each antenna (left antenna measured; FL 0.18mm; CL 0.18mm; CL/FL 1.00) with length of antennomeres (in mm) as follows: 0.08; 0.06; 0.06; 0.05; 0.03; 0.02; 0.02; 0.05; 0.05; 0.08. Pronotum with single and coarse punctation; punctures very close to each other on disc and near the lateral and posterior margins, but somewhat shallower and separated by a distance of about one puncture-width at the anterior projection; in between punctures finely granulate; vestiture grayish, dual (seen in lateral view under a magnification of 100×), consisting of conspicuous stout erect bristles (~0.05mm) and very small decumbent setae (a bit less than 0.02mm); anterior angles rounded, produced forward; anterior edge explanate and produced forward forming a plate that slightly curves upward and tapers toward a rounded apex; lateral margins slightly explanate and visible for their entire lengths from above, irregularly crenulate. Hindwings fully developed. Scutellum subtriangular, densely covered by stout decumbent bristles (better seen in lateral view); basal width 0.15mm. Sides of elytra parallel at basal three-fourths, broadly rounded posteriorly (seen from above) and converging to a blunt apex; punctation single and confused; punctures coarse and separated by a distance of one puncture-width or less; in between punctures dull; vestiture dual, similar to that of pronotum; lateral and apical margins not visible from above; epipleura tapering from base to middle, then enlarging to apex, with external margin simple (not crenulate). Ventral surface of thorax and abdomen finely granulate, with vestiture of decumbent slender setae. Prosternum flat; prosternal process laminate, three-fourths the length of procoxae. First abdominal ventrite twice as long as the second at midline; setose sex patch circular and margined, located near the posterior margin and with a diameter of one-fifth the length of the ventrite at midline.

**Male terminalia in paratypes (Figs 5–6).** Eighth sternite (Fig. 5) with posterior margin straight; posterior angles slightly produced forming two small lateral prominences. Basal piece (Fig. 6) nearly one-third the length of tegmen. Tegmen (Fig. 6) with anterior portion subtriangular; lateral margins straight, slightly diverging to apex; posterior portion with a median V-shaped emargination of about one-third the length of tegmen, forming two lateral lobes. Penis (Fig. 6) as long as tegmen, subcylindrical; lateral margins straight for half of their lengths then slightly curved inwards to a narrowly rounded apex.

**Females (Fig. 4).** Similar to males, but frontoclypeal ridge straight, barely sinuous, with rounded angles. Anterior pronotal margin broadly rounded. Abdomen devoid of sex patch.

#### Variation.

**Figures 7–9.**
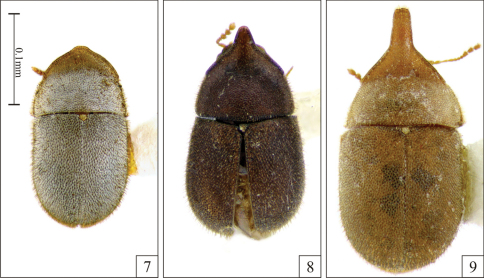
*Falsocis aquilonius* Lopes-Andrade & Lawrence, sp. n., male paratypes, dorsal view, shown in the same scale. **7** Low male from Cerro Campana (Panama) **8** Medium male from La Lola (Costa Rica) **9** High male from Puntarenas (Costa Rica).

Males, measurements in mm (n = 6, including the holotype): TL 2.00–2.84 (2.45 ± 0.32); PL 0.74–1.32 (1.02 ± 0.20); PW 1.00–1.37 (1.16 ± 0.12); EL 1.21–1.47 (1.37 ± 0.11); EW 1.05–1.42 (1.23 ± 0.12); GD 0.84–1.16 (0.97 ± 0.13). Ratios: PL/PW 0.74–0.96 (0.87 ± 0.09); EL/EW 1.04–1.17 (1.12 ± 0.05); EL/PL 1.12–1.64 (1.37 ± 0.18); GD/EW 0.74–0.88 (0.79 ± 0.05); TL/EW 1.90–2.17 (1.99 ± 0.10). The anterior plate (projected anterior margin) of pronotum in males varies from very small (Fig. 7), medium size (Fig. 8, similar to that of holotype) to extremely projected (Fig. 9); but the apex of this plate is rounded or blunt and devoid of tufts. Females, measurements in mm (n = 5): TL 2.21–2.53 (2.38 ± 0.14); PL 0.79–0.89 (0.83 ± 0.04); PW 1.11–1.37 (1.24 ± 0.10); EL 1.37–1.63 (1.47 ± 0.11); EW 1.16–1.47 (1.33 ± 0.13); GD 0.95–1.16 (1.05 ± 0.08). Ratios: PL/PW 0.60–0.81 (0.67 ± 0.08); EL/EW 1.04–1.27 (1.12 ± 0.09); EL/PL 1.63–1.94 (1.78 ± 0.15); GD/EW 0.74–0.95 (0.80 ± 0.09); TL/EW 1.68–2.14 (1.81 ± 0.19).

#### Type series.

*Holotype.* (ANIC) **Panama:** \R.P.: Panama Cerro Campana Feb. 22, 1975 Lawrence, Erwin \ *Phellinus* sp. \ J.F. Lawrence Lot [printed] 3799 [handwritten] \ *Falsocis* *aquilonius* Lopes-Andrade & Lawrence HOLOTYPUS [printed on red paper]\. *Paratypes.* **Panama:** 1 male and 1 female (ANIC), same data as holotype; 3 males (1 ANIC, 2 LAPC), 4 females (2 ANIC, 1 LAPC) \2.5mi S.W.Rincon Puntarenas, Costa Rica, Mar. 1–7, ’67 \ J.F. Lawrence Lot. [printed] 2175 [handwritten] \ R. Andrews Collector\. **Costa Rica.** 1 male (ANIC) \La Lola, C.R. III-8–1958 M.J. Stelzer [handwritten] \ MS 58.4 [handwritten]\; 1 female (ANIC) \Puerto Viejo COSTA RICA VIII-4–65 \ J.F. Lawrence Lot [printed] 1611 [handwritten]\. **Colombia.** 1 male (Marseul Collection, MNHN) \Dupont Colomb [18]41 [handwritten in circular green paper] \ MARSEUL [handwritten in rectangular small green paper] \ [circular blue paper, without information] \ FALSOCIS SPP. [handwritten] det. J.F. Lawrence 19 [printed]\. All paratypes distinguished labeled \*Falsocis* *aquilonius* Lopes-Andrade & Lawrence PARATYPUS [printed on yellow paper]\.

#### Comments and comparative notes.

One specimen collected in an unidentified *Phellinus*. This is the only *Falsocis* species known from Central America, but it also occurs at the northernmost South America (Fig. 41). Recent field collections in Colombia were unsuccessful in recapturing the species, so the country record is based only on a single old male without precise locality data. In *Falsocis opacus*, the epipleura is much more enlarged, easily visible from above, with external margin crenulate; the male eighth sternite is similar, but basal piece is strongly developed and is almost half the length of aedeagus; tegmen is elongate, four times as long as wide. In the remaining *Falsocis*, the epipleura is narrower near the apex.

### Falsocis egregius

Lopes-Andrade & Lawrence sp. n.

urn:lsid:zoobank.org:act:E6BFE936-A896-449F-8D6A-ADE8528F8728

http://species-id.net/wiki/Falsocis_egregius

Figs 10–16 41

#### Type locality.

Santarém, in the state of Pará, northern Brazil (2°26'S, 54°42'W).

#### Etymology.

The specific epithet is from the Latin “egregius” (adjective), which means “singular”, “extraordinary”, in a reference to the outstanding head morphology of males.

#### Diagnosis.

**Figures 10–16.**
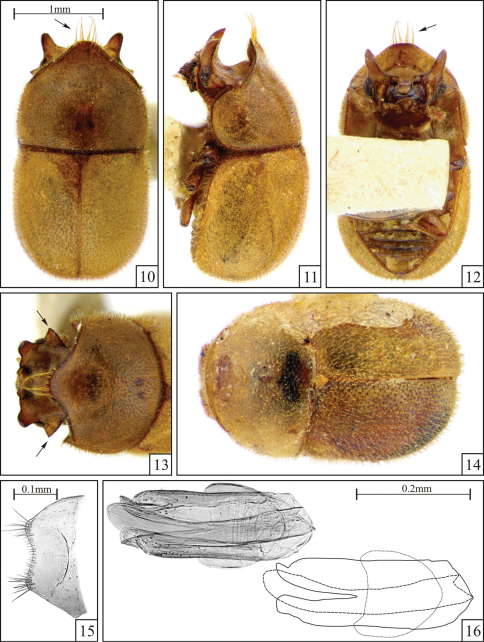
*Falsocis egregius* Lopes-Andrade & Lawrence, sp. n., male holotype **10–13** and female paratype **14** shown in the same scale, and slide preparation of male terminalia of a paratype **15–16**. **10** Dorsal view (pronotal tuft of long setae, arrow) **11** Lateral view **12** Ventral view (pronotal tuft of long setae, arrow) **13** Head and pronotum view from above (triangular plate behind eyes, arrows) **14** Female **15** Eighth sternite **16** Aedeagus (left) and gross outline of aedeagus (right) showing tegmen (continuous line), penis (dashed line) and basal piece (dotted line).

Head of male with sides of vertex produced laterally to form a pair of triangular plates, each with acute apex (Fig. 13, arrows); frontoclypeal horns wide at base and tapering to apex, slightly arcuate. Pronotum with anterior angles broadly rounded, barely produced forward; males with a row of long setae along the apex of the anterior projection but not forming distinct tufts (Figs 10, 12, arrows).

#### Description.

Male holotype (Figs 10-13), measurements in mm: TL 2.85; PL 1.25; PW 1.50; EL 1.55; EW 1.55; GD 1.35. Ratios: PL/PW 0.83; EL/EW 1.00; EL/PL 1.24; GD/EW 0.87; TL/EW 1.84. Body oblong, strongly convex, mostly light yellowish brown; mouthparts and apices of femora reddish brown; ventral vestiture consisting of very fine decumbent setae. Head strongly developed, the anterior and lateral margins easily visible from above; dorsum concave, slightly tumid on disc; punctation relatively fine, shallow, sparse; in between punctures finely granulate; frontoclypeal ridge explanate, each side produced upward forming a conspicuous horn that is slightly arcuate, bearing two small tubercles between them; each side of vertex explanate and produced laterally forming a conspicuous triangular plate with an acute apex (Fig. 13, arrows). Eyes coarsely facetted; greatest width 0.18mm. Each antenna (left antenna measured; FL 0.22mm; CL 0.23mm; CL/FL 1.05) with length of antennomeres (in mm) as follows: 0.12; 0.07; 0.08; 0.04; 0.04; 0.03; 0.03; 0.06; 0.06; 0.11. Pronotum with shallow, coarse, single punctation; punctures separated by a distance about a puncture-width; in between punctures smooth at disc and finely granulate at the anterior projection; vestiture indistinctly dual, consisting of conspicuous yellowish stout erect bristles (~0.05mm) and very fine decumbent setae (<0.02mm); anterior angles broadly rounded, just slightly produced forward; anterior margin (beyond anterior angles) explanate, produced forward forming a plate that slightly curves downward and narrows toward a slightly arcuate apex with a row of very long slender setae (>0.35mm; Figs 10, 12, arrows). Scutellum subtriangular; punctation finer than those of pronotum and elytra; vestiture consisting of stout decumbent bristles (better seen in lateral view); basal width 0.21mm. Hindwings fully developed. Elytra subparallel at basal two-thirds, with posterolateral angles broadly rounded (as seen from above) and then converging to a blunt apex; punctation single and confused, a bit sparser than that of pronotum; in between punctures smooth; vestiture indistinctly dual, consisting of conspicuous stout erect bristles (~0.07mm) and very minute decumbent setae (<0.02mm); lateral and apical margins not visible from above; epipleura tapering from base to apex. Surface of the ventral thoracic and abdominal sclerites granulate, somewhat rugose. Prosternum flat; prosternal process laminate, almost half the length of procoxae. First abdominal ventrite more than twice as long as the second at midline; setose sex patch circular and margined, located at the middle of the ventrite and with a diameter of one-third the length of the ventrite at midline.

**Male terminalia in paratypes (Figs 15–16).** Eighth sternite (Fig. 15) with posterior margin curved inward; angles not produced. Basal piece (Fig. 16) almost half the length of tegmen. Tegmen (Fig. 16) with anterior portion broadly rounded; lateral margins almost straight; posterior portion bearing a deep V-shaped emargination reaching the middle of the structure and forming two lateral lobes. Penis (Fig. 16) subcylindrical, lateral margins straight for most of their lengths and a bit expanded to a rounded apex; the penis is turned and lays laterally in the slide preparation.

**Female (Fig. 14).**Similar to males, but frontoclypeal ridge straight, barely sinuous, with rounded angles. Anterior pronotal margin broadly rounded. Abdomen devoid of sex patch.

#### Variation.

Males, measurements in mm (n = 5, including the holotype): TL 2.75–2.95 (2.87 ± 0.08); PL 1.20–1.35 (1.26 ± 0.05); PW 1.50–1.55 (1.52 ± 0.03); EL 1.50–1.65 (1.56 ± 0.05); EW 1.55–1.65 (1.57 ± 0.04); GD 1.30–1.40 (1.35 ± 0.04). Ratios: PL/PW 0.80–0.87 (0.83 ± 0.03); EL/EW 0.97–1.00 (0.99 ± 0.01); EL/PL 1.15–1.32 (1.24 ± 0.06); GD/EW 0.84–0.87 (0.86 ± 0.02); TL/EW 1.77–1.87 (1.83 ± 0.05).

Female, measurements in mm (n = 1): TL 2.50; PL 0.95; PW 1.45; EL 1.55; EW 1.50; GD 1.05. Ratios: PL/PW 0.66; EL/EW 1.03; EL/PL 1.63; GD/EW 0.70; TL/EW 1.67.

#### Type series.

*Holotype.* (CMNH) **Brazil:** \Santarem Brazil Acc. No.2966 \ CM \ *Falsocis* *egregius* Lopes-Andrade & Lawrence HOLOTYPUS [printed on red paper]\. *Paratypes.* **Brazil:** 4 males (1 ANIC; 2 CMNH; 1 LAPC, dissected) and 1 female (CMNH) \Santarem Brazil Acc. No.2966 \ CM\, same data as holotype. All paratypes distinguished labeled \*Falsocis egregius* Lopes-Andrade & Lawrence PARATYPUS [printed on yellow paper]\.

#### Comments and comparative notes.

The species is currently known from a single collection in Santarém, northern Brazil (Fig. 41). Only one female was available for examination. The series is very small, but species sufficiently distinct to allow description. Differs from *Falsocis aquilonius* sp. n. and *Falsocis opacus* in having the epipleura narrow posteriorly. *Falsocis occultus* sp. n. has deeper and closer pronotal punctation. Very similar to *Falsocis brasiliensis*, but differing mainly in features mentioned in the diagnosis and in the conspicuous basal piece of male terminalia. In *Falsocis brasiliensis*, the eighth sternite has the anterior margin only slightly curved inward; the basal piece was not observed in the available slide preparations and is possibly membranous (Lopes-Andrade 2007); the tegmen has a subtriangular posterior portion, subparallel lateral margins and anterior V-shape emargination is only one-fourth to one-third deep.

### Falsocis occultus

Lopes-Andrade & Lawrence sp. n.

urn:lsid:zoobank.org:act:EF30CDE6-0A8A-444A-9A89-0ECB68E9CB17

http://species-id.net/wiki/Falsocis_occultus

Figs 17 –25 41

#### Type locality.

Linhares, in the state of Espírito Santo, southeastern Brazil (19°23'S, 40°04'W).

#### Etymology.

The specific epithet is from the Latin “occultus” (adjective), which means “hidden”, in reference to the fact that the population from the type locality stayed hidden among fungi forgotten in a field-base for near four years.

#### Diagnosis.

Pronotum with anterolateral angles not produced forward; lateral margins not visible from above; male with anterior projection ending in an acute apex (Figs 19, 23-25, arrows) bordered by medium-size bristles.

#### Description.

**Figures 17–22.**
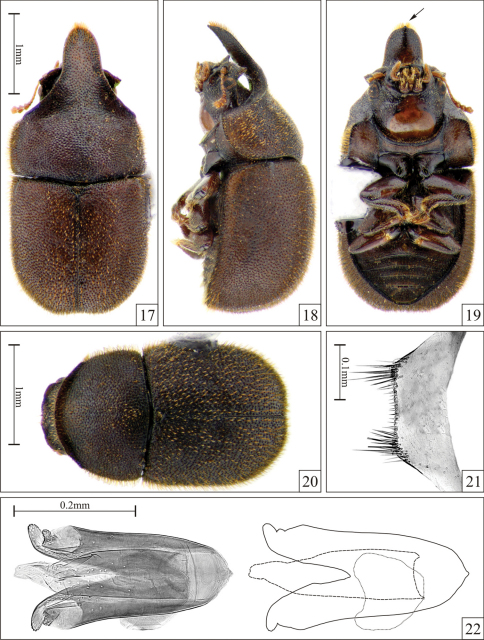
*Falsocis occultus* Lopes-Andrade & Lawrence, sp. n., male holotype **17–19** and female paratype **20** shown in the same scale, and slide preparation of male terminalia of a paratype **21–22**. **17** Dorsal view **18** Lateral view **19** Ventral view (acute pronotal apex, arrow) **20** Dorsal view **21** Eighth sternite **22** Aedeagus (left) and gross outline of aedeagus (right) showing tegmen (continuous line), penis (dashed line) and basal piece (dotted line).

Male holotype (Figs 17–19), measurements in mm: TL 3.55; PL 1.70; PW 1.75; EL 1.80; EW 1.75; GD 1.40. Ratios: PL/PW 0.97; EL/EW 1.03; EL/PL 1.06; GD/EW 0.80; TL/EW 2.03. Body oblong, strongly convex, mostly dark brown; mouthparts, antennae and tarsi dark yellowish brown; femora and tibiae dark reddish brown. Head concealed by the anterior pronotal projection (seen from above) except for its anterolateral angles; dorsum concave with disc slightly tumid; punctation coarse, shallow; in between punctures finely granulate; frontoclypeal ridge explanate and produced forming a broad acute triangular plate at each anterior angle, with two small tubercles between them. Eyes coarsely facetted; greatest eye width 0.21mm. Each antenna (left antenna measured; FL 0.30mm; CL 0.28mm; CL/FL 0.93) with length of antennomeres (in mm) as follows: 0.15; 0.09; 0.08; 0.08; 0.06; 0.05; 0.03; 0.08; 0.08; 0.12. Pronotum with single, coarse, relatively deep punctation; punctures very close to each other, separated by a distance of one puncture-width or less; in between punctures smooth but not shining; vestiture yellowish, indistinctly dual (seen under a magnification of 100×), consisting of stout erect bristles (~0.05mm) and small decumbent setae (~0.03mm), both better seen in lateral view; anterior angles not produced forward; anterior margin (beyond anterior angles) explanate, strongly produced forward forming a plate that slightly curves downward and narrows toward an acute apex (Fig. 19, arrow) ornamented by a row of increasingly stout bristles in either side (Figs 17-19); lateral margins slightly crenulate, not visible from above, bearing a row of stout bristles. Scutellum subtriangular, its margins indistinct so that it seems to be contiguous with elytra; punctation conspicuous but slightly finer than those of pronotum and elytra; vestiture consisting of stout decumbent bristles (better seen in lateral view); basal width 0.20mm. Hindwings fully developed. Elytra parallel at basal three-fourths, posteriorly broadly rounded (as seen from above) and converging to a blunt apex; punctation single and confused, slightly finer than that of pronotum, consisting of relatively deep punctures separated by a distance near a puncture-width; in between punctures smooth, dull; vestiture distinctly dual, the erect bristles about 0.1mm long and the decumbent setae about 0.03mm long; lateral and apical margins not visible from above; epipleura tapering from base to the basal one-sixth, then continuing as a narrow line to the apex. Ventral surfaces of thorax and abdomen finely granulate; vestiture consisting of slender decumbent setae. Prosternum flat; prosternal process laminate, two-thirds the length of the procoxae. First abdominal ventrite more than twice as long as the second at midline; setose sex patch suboval and margined, located at the middle of the ventrite and with a diameter of one-third the length of the ventrite at midline.

**Male terminalia in paratypes (Figs 21–22).** Eighth sternite (Fig. 21) with posterior margin almost straight; angles not produced. Basal piece (Fig. 22) nearly one-third the length of tegmen. Tegmen (Fig. 22) with anterior portion mostly rounded but apex acute; lateral margins slightly sinuous and diverging; posterior portion bearing a deep V-shaped emargination reaching the middle of the structure, forming two lateral lobes. Penis (Fig. 22) subcylindrical; lateral margins subparallel for most of their lengths; apical third subtriangular, membranous.

**Females (Fig. 20).** Similar to males, but frontoclypeal ridge straight, barely sinuous, with rounded angles. Anterior pronotal margin broadly rounded. Abdomen devoid of sex patch.

#### Variation.

**Figures 23–25.**
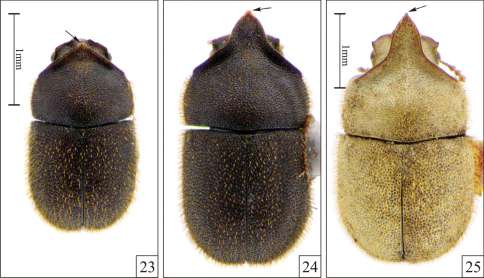
*Falsocis occultus* Lopes-Andrade & Lawrence, sp. n., male paratypes **23–24** shown in the same scale, dorsal view (acute pronotal apex, arrows). **23** Low male from Linhares (in the state of Espírito Santo, southeastern Brazil) **24** Medium male from Linhares **25** The unique specimen known from Nova Teutônia (in the state of Santa Catarina, southern Brazil).

Males, measurements in mm (n = 15, including the holotype): TL 2.11–3.55 (2.84 ± 0.42); PL 0.84–1.70 (1.28 ± 0.25); PW 1.16–1.75 (1.48 ± 0.17); EL 1.25–1.80 (1.53 ± 0.19); EW 1.21–1.84 (1.52 ± 0.17); GD 0.95–1.47 (1.22 ± 0.16). Ratios: PL/PW 0.73–0.97 (0.86 ± 0.07); EL/EW 0.86–1.07 (1.01 ± 0.05); EL/PL 1.00–1.50 (1.22 ± 0.15); GD/EW 0.72–0.93 (0.80 ± 0.05); TL/EW 1.73–2.03 (1.86 ± 0.09). In the unique specimen from Nova Teutonia, a teneral male, pronotal and elytral bristles are larger (0.08mm and 0.15mm, respectively) than in specimens from Linhares. In small males, the anterior pronotal plate is barely projected (Fig. 23). However, all males have an acute pronotal apex (Figs 19, 23–25, arrows). Females, measurements in mm (n = 15): TL 2.30–3.15 (2.63 ± 0.27); PL 0.80–1.16 (0.97 ± 0.11); PW 1.05–1.75 (1.42 ± 0.19); EL 1.40–2.00 (1.64 ± 0.18); EW 1.10–1.85 (1.50 ± 0.19); GD 1.05–1.50 (1.23 ± 0.15). Ratios: PL/PW 0.62–0.81 (0.69 ± 0.05); EL/EW 1.00–1.41 (1.11 ± 0.10); EL/PL 1.50–2.00 (1.71 ± 0.15); GD/EW 0.79–0.95 (0.83 ± 0.04); TL/EW 1.64–2.23 (1.77 ± 0.14).

#### Type series.

*Holotype.* (LAPC) **Brazil:** \BRASIL: ES Linhares 11–21.x.2004 P.C. Grossi leg. \ *Falsocis* *occultus* Lopes-Andrade & Lawrence HOLOTYPUS [printed on red paper]\. *Paratypes.* **Brazil:** 56 (27 males, 1 dissected, and 19 females, LAPC; 5 males and 5 females, ANIC), same data as holotype; 1 male (FMNH) \Nova Teutonia, Sta. Catharina, BRAZ. 300–500m alt. Fritz Plaumann leg [printed] XI:1940 [handwritten] \ [circular red paper, without information] \ Falsocis sp. 115 \. All paratypes distinguished labeled \*Falsocis occultus* Lopes-Andrade & Lawrence PARATYPUS [printed on yellow paper]\.

#### Other specimens examined.

26 specimens, gender not determined (2 CNCI for molecular analysis, 4 LAPC in absolute alcohol, 20 MTD), same data as holotype.

#### Comments and comparative notes.

**Figures 26–31.**
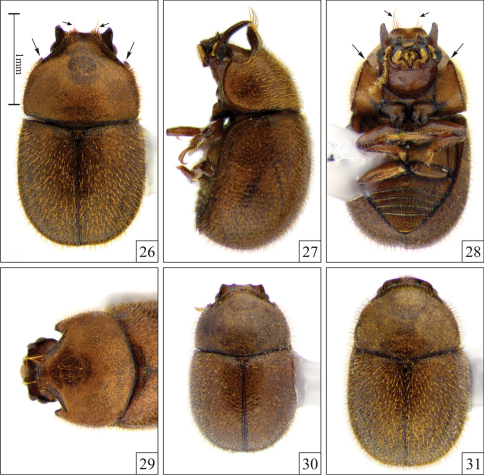
*Falsocis brasiliensis* Lopes-Andrade, 2007, specimens from Viçosa (in the state of Minas Gerais, southeastern Brazil), all shown in the same scale. **26** Dorsal view (produced angles, large arrows; two tufts of long setae, small arrows) **27** Lateral view **28** Ventral view (produced angles, large arrows; two tufts of long setae, small arrows) **29** Head and pronotum view from above **30** Low male **31** Female.

Known from two localities, in southeastern and southern Brazil (Fig. 41). The specimens from Linhares (in the state of Espírito Santo, southeastern Brazil) were collected in *Hexagonia papyracea* Berk.(Polyporaceae) and bred in the laboratory until December 2009 in the original basidiomes, without addition of either water or nutrients. In male *Falsocis brasiliensis* and *Falsocis egregius* sp. n., the apex of the pronotal projection bears a row of setae that are comparatively longer (Figs 10–12, 26–29, 33–34). Differs from *Falsocis aquilonius* sp. n. and *Falsocis opacus* in having the epipleura narrow posteriorly.

### New distributional data, additional comments and comparative notes

#### Falsocis brasiliensis

Lopes-Andrade, 2007

http://species-id.net/wiki/Falsocis_brasiliensis

Figs 26 –34 41

##### Additional records.

**Brazil:** 1 female (LAPC) \BRASIL: ES Santa Maria de Jetibá 08.xii.2003 leg. Furieri & Nunes\; 3 males (1 LAPC, in absolute alcohol; 2 CNCI, for molecular analysis) and 1 female (LAPC, in absolute alcohol) \BRASIL: MG Viçosa “Belvedere” 03.ii.2011 leg. L.S. Araújo, C.A. Carvalho\; 2 males and 1 female (LAPC) \BRASIL: MG Viçosa Mata da Biologia, ponto 37 16.iv.2010; leg. T. Mariani & C. Lopes-Andrade; ex *Hymenochaete luteobadia*\; 2 males (LAPC) \BRASIL: MG Viçosa Mata da Biologia, ponto 048 20.iv.2011; C. A. Carvalho & C. Lopes-Andrade; ex *Hymenochaete luteobadia*\.

##### Comments.

**Figures 32–34.**
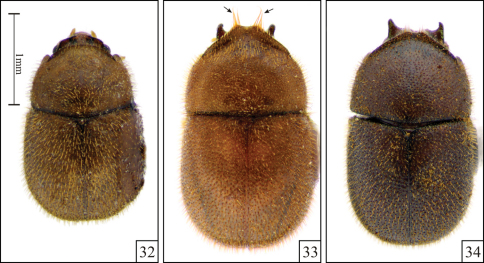
*Falsocis brasiliensis* Lopes-Andrade, 2007, dorsal view, shown in the same scale. **32** The unique female specimen from Santa Maria de Jetibá, in the state of Espírito Santo, southeastern Brazil **33** Male paratype from Venda Nova do Imigrante, Espírito Santo (two tufts of long setae, arrows) **34** Male paratype from Jussari (in the state of Bahia).

The specimens from Viçosa (in the state of Minas Gerais, southeastern Brazil; Figs 26–31) were all collected in *Hymenochaete luteobadia* (Fr.) Höhn. & Litsch. (Hymenochaetacea). This fungus was previously misidentified as *Phellinus* sp. (Lopes-Andrade 2007, Graf-Peters et al. 2011). After its description, the species was recollected in Viçosa and a single female was found in Santa Maria de Jetibá (in the state of Espírito Santo, southeastern Brazil; Fig. 32). The species is restricted to forests, known only from small remnants, and was never found in open areas. Specimens from Venda Nova do Imigrante (Espírito Santo; Fig. 33) are reddish, and the ones from Jussari (in the state of Bahia; Fig. 34) are dark brown. In low males (Fig. 30), the frontoclypeal and pronotal projections are very weak and the pronotum narrower anteriorly. TL (in mm, not including the head) and TL/EW of the type series are as follows: Males (n = 10, including the holotype), TL 2.00–2.80 (2.25 ± 0.22), TL/EW 1.54–1.81 (1.64 ± 0.07); Females (n = 5), TL 2.00–2.35 (2.14 ± 0.14), TL/EW 1.48–1.68 (1.60 ± 0.08).

#### Falsocis opacus

Pic, 1916

http://species-id.net/wiki/Falsocis_opacus

Figs 35–37 41

##### Comments.

The subspecies *Falsocis opacus flavus* Pic, 1922 was based on a single female collected in Bas-Maroni (French Guyana, near Cayenne), and it is merely a teneral specimen of *Falsocis opacus* (J. F. Lawrence pers. obs.).The type of *Falsocis opacus* (MNHN) is 2.10mm long, rather than 3mm as mentioned in the description (Pic 1916), but other measurements were not taken by the time it was examined. In the unique male *Falsocis opacus* measured by Lopes-Andrade (2007), TL is 3.05mm and TL/EW 1.91, not including the head.

#### Falsocis sp.

Figs 38- 41

##### Record.

1 female (MNHN) **Peru:** \Mission de Sarayacu (Riv. Oucayale) [Ucayali] [handwritten in green paper] \ CO 47 [handwritten in a circular yellowish paper] \ MUSEUM PARIS PÉROU PAMPAS [sic] DEL SACRAMENTO DE CASTELNAU 1847 [written in light brown paper] \ [circular blue paper, without information]\.

##### Comments and comparative notes.

The specimen was collected at Sarayacu Mission (6°47'S, 75°07'W, Fig. 41) during the French Scientific Expedition to the Pampa del Sacramento, Peru, in 1847. It resembles females of *Falsocis brasiliensis*, *Falsocis egregius* sp. n. and *Falsocis occultus* sp. n. in having the epipleura narrow posteriorly. However, the shallow punctation of pronotum resembles mostly that of the former two species. Female *Falsocis brasiliensis* from Jussari (Brazil) are similar in color, but body is more elongate. It is very large, with a length of near 3mm. Only females of *Falsocis occultus* sp. n. reach such a length. A subquadrate body is also observed in a few *Falsocis brasiliensis*, but the largest females of this species are only 2.4mm long. The very transverse pronotum, twice as long as wide, does not occur in any other known *Falsocis* species. This female from Peru possibly belongs to another new species, but description is not possible without examining a male. Measurements (in mm) and ratios are as follows: TL 3.05; PL 1.00; PW 2.00; EL 2.00; EW 2.11; GD 1.68; PL/PW 0.50; EL/EW 0.95; EL/PL 2.00; GD/EW 0.80; TL/EW 1.45.

**Figures 35–40.**
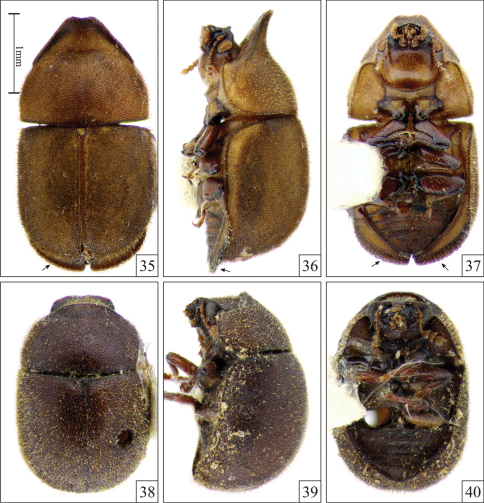
*Falsocis opacus* Pic, 1916, from Altamira, in the state of Pará, northern Brazil **35–37**; arrows pointing out the explanate outer apical margins of epipleura and *Falsocis* sp., female from Peru **38–40**, all shown in the same scale. **35, 38** Dorsal view **36, 39** Lateral view **37, 40** Ventral view.

**Figure 41.**
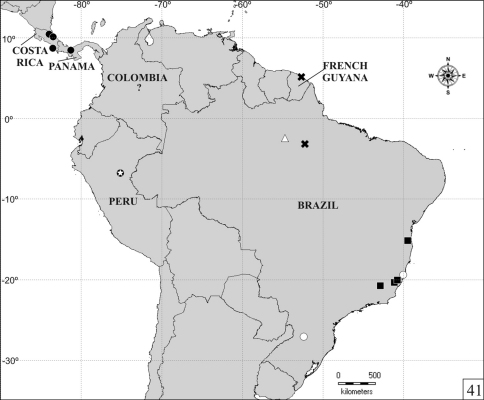
Distribution map for *Falsocis* Pic species. *Falsocis aquilonius* sp. n. (full circle), *Falsocis brasiliensis* Lopes-Andrade (full squares), *Falsocis egregius* sp. n. (open triangle), *Falsocis occultus* sp. n. (open circles), *Falsocis opacus* Pic (x) and *Falsocis* sp. (open star inside full circle). The question mark indicates an imprecise location of *Falsocis aquilonius* sp. n. in Colombia.

**Identification key to the species of *Falsocis* Pic**

**Table:** 

1	Epipleura enlarged posteriorly, forming a slightly or strongly explanate posterolateral margin in elytra, conspicuously or barely visible from above	2
1’	Epipleura narrowing to apex, forming a narrow, not explanate, posterolateral margin in elytra, not visible from above	3
2(1)	Pronotum with lateral margins visible from above. Posterior elytral margin (outer margin of epipleura) simple (not crenulate), barely visible from above. Known from Panamá, Costa Rica and Colombia	*Falsocis aquilonius* sp. n.
2’	Pronotum with lateral margins not visible from above. Posterior elytral margin (outer margin of epipleura) crenulate, easily visible from below and above (Figs 35–37, arrows). Known from Brazil (Altamira, in the state of Pará) and French Guyana	*Falsocis opacus* Pic, 1916
3(1’)	Pronotal punctation deep and coarse, with punctures usually separated by half a puncture-width or less at disc. Anterior plate of pronotum in males devoid of tufts of very long setae and acute at apex (Figs 19, 23–25)	*Falsocis occultus* sp. n.
3’	Pronotal punctation shallow and fine, with punctures usually separated by about a puncture-width at disc. Anterior plate of pronotum in males with slender setae usually as long as to twice as long (or more) than an eye-width, with apex straight, barely emarginated or slightly rounded	4
4(3’)	Anterior pronotal angles distinctly produced forward and somewhat acute (Figs 26, 28, large arrows). Males with head devoid of conspicuous projection behind each eye (Fig. 29); long setae of anterior pronotal plate organized in two tufts (Figs 26, 28, 33, small arrows)	*Falsocis brasiliensis* Lopes-Andrade, 2007
4’	Anterior pronotal angles barely produced forward and rounded (Figs 10-12). Males with head bearing a conspicuous triangular plate, projected outward, behind each eye (Fig. 13, arrows); long setae of anterior pronotal plate organized as a row, not forming tufts (Figs 10, 12, arrows)	*Falsocis egregius* sp. n.

## Discussion

The phylogenetic position of *Falsocis* is uncertain. In the available phylogenetic analysis of the family, based on molecular data, *Falsocis brasiliensis* was not part of any defined clade (Buder et al. 2008). It is morphologically related to *Acanthocis* Miyatake from Japan, with a unique combination of features among Ciini (Lopes-Andrade 2007): prominent outer apical angle of protibiae, forming an acute tooth; laminate prosternal process; and subconical procoxae. However, these features are also observed in *Porculus* Lawrence, in which species also have a very elongate female ovipositor similar to that of *Falsocis*, male terminalia with tegmen bearing a deep V-shaped emargination and subcylindrical penis (Lopes-Andrade pers. obs). *Porculus* differs from *Falsocis* and *Acanthocis* in the single punctation of pronotum and elytra, and dorsal vestiture consisting of minute setae (Lawrence 1987, Lopes-Andrade 2007). A strongly sclerotized female ovipositor devoid of gonostyli was observed in *Porculus grossus* Lawrence (Lawrence and Lopes-Andrade 2010), but in *Porculus brunneus* (Mellié) the structure is complete and less sclerotized, similar to that of *Falsocis* species. *Falsocis* and *Porculus* are restricted to the Neotropical region (sensu Morrone 2002), and *Acanthocis* is known only from Japan (Lawrence and Lopes-Andrade 2010). These three genera possibly constitute an independent lineage of Ciinae.

Species of *Falsocis* are not frequently collected and all are allopatric. They are known only from forests or forest remnants and usually one or few specimens are found together in the field. The greatest series is that for *Falsocis occultus* sp. n., which was reared in laboratory. Accurate data on host fungi are scarce. Available information on *Falsocis brasiliensis* suggests the species is monophagous (Graf-Peters et al. 2011). It was considered an endangered species by Lopes-Andrade (2007). However, not only this species but possibly all *Falsocis* are seriously threatened or endangered. For instance, besides great collection efforts in the state of Rio Grande do Sul, southern Brazil (Graf-Peters et al. 2011) and in an unpublished survey in Pará, northern Brazil (Lopes-Andrade pers. obs.), no *Falsocis* was collected. Most of the recent records (from 2000 up to know) are from the Brazilian Atlantic Forest, from small remnants in southeastern and the most southern portion of northeastern Brazil. The single old record of *Falsocis occultus* sp. n. from Nova Teutônia, in the state of Santa Catarina, suggests the species would have had a very broad distribution throughout the Atlantic Forest. This biome has been seriously affected and fragmented by land use and urbanization, and less than 8% of the original forests still remain (Colombo and Joly 2010).

## Supplementary Material

XML Treatment for Falsocis aquilonius

XML Treatment for Falsocis egregius

XML Treatment for Falsocis occultus

XML Treatment for Falsocis brasiliensis

XML Treatment for Falsocis opacus

XML Treatment for Falsocis
